# Effects of aerosols from heated tobacco products with flavors on the discoloration of bovine tooth enamel

**DOI:** 10.1002/cre2.764

**Published:** 2023-07-12

**Authors:** Takeshi Kurachi, Shinnosuke Chuman, Takuya Suzuki, Terushige Kubota, Shinkichi Ishikawa

**Affiliations:** ^1^ Scientific Product Assessment Center, R&D Group, Japan Tobacco Inc. Yokohama Kanagawa Japan; ^2^ Scientific and Regulatory Affairs, JT International SA Geneva Switzerland; ^3^ Scientific and Regulatory Affairs, Japan Tobacco Inc. Tokyo Japan

**Keywords:** dental enamel, in vitro techniques, smoking, tooth discoloration

## Abstract

**Objectives:**

This in vitro study assessed the potential of tooth discoloration by aerosols generated from three heated tobacco products (HTPs) with different specifications: in‐direct heating tobacco system platform 1.0a (IT1.0a), in‐direct heating tobacco system platform 2.0a (IT2.0a), and direct heating tobacco system platform 3.0a (DT3.0a). In addition, three flavor types (regular, menthol, and berry menthol) were selected for each HTP to characterize the effect of flavor types on tooth discoloration.

**Material and Methods:**

Six bovine tooth samples were exposed directly to aerosols generated from one pack of each HTP: 350 puffs for IT1.0a, 325 puffs for IT2.0a, and 220 puffs for DT3.0a. Six bovine tooth samples were also exposed to air (350 puffs) and smoke generated from one pack of cigarettes (160 puffs) as negative and positive controls, respectively. The color of each tooth sample was measured before and after exposure. The overall color changes were assessed using overall color differences (Δ*E*) calculated according to the Commission International de I'Eclairage color system. A one‐way analysis of variance followed by Tukey's post hoc test was used to compare Δ*E* among bovine tooth samples exposed to air, cigarette smoke, and aerosols generated from each HTP.

**Results:**

Δ*E* values for tooth samples exposed to air and aerosols generated from the three HTPs (IT1.0a, IT2.0a, and DT3.0a) were significantly lower than Δ*E* value for tooth samples exposed to cigarette smoke. Δ*E* values obtained with DT3.0a were significantly higher than those obtained with air‐exposed control samples. However, Δ*E* values obtained with IT1.0a and IT2.0a were not significantly different from that obtained with air‐exposed control samples. No HTPs showed significant differences in Δ*E* values among the three flavor types.

**Conclusions:**

This study showed that HTP aerosols reduce tooth discoloration potential compared with cigarette smoke, regardless of flavor types, and the tooth discoloration potential of the product may depend on product specifications.

## 1 INTRODUCTION

Next‐generation tobacco products are gaining popularity as a potential alternative to conventional cigarettes. Among the products categorized as next‐generation tobacco products, e‐cigarettes and heated tobacco products (HTPs) are particularly popular. HTPs are designed to generate aerosols by heating tobacco rather than burning it. Although the heating temperature of HTPs depends on the product specifications, it is usually designed to be below 350°C, which is lower than the burning temperature of conventional cigarettes (700–950°C) (Mallock et al., 2019). Therefore, HTPs should generate reduced levels of harmful and potentially harmful constituents (HPHCs) that are derived from the combustion of tobacco leaves and found in cigarette smoke (Mallock et al., 2019). Previous studies indicated that aerosols from several HTPs have lower levels of HPHCs (Forster et al., 2018; Schaller et al., 2016; Takahashi et al., 2018) and the biological effects are reduced in in vitro (Ishikawa et al., 2018; Ito et al., 2020; Munakata et al., 2018; Schaller et al., 2016; Thorne et al., 2018), in vivo (Phillips et al., 2016), and clinical (Gale et al., 2021; Haziza et al., 2020; Sakaguchi et al., 2021) studies when compared with conventional cigarette smoke. These study data support the fact that HTPs have the potential to reduce the health risks associated with smoking.

Concerns associated with smoking are not limited to health risks. White teeth are generally considered aesthetically pleasing in various societies and cultures (Afroz et al., 2013; Bersezio et al., 2018; Khalid & Quiñonez, 2015). However, along with food, beverages, and drugs, smoking is also associated with tooth discoloration (Joiner et al., 2008; Watts & Addy, 2001). The exact mechanism behind tooth discoloration caused by smoking has not been fully identified. However, tooth surface deposition of brown pigments from the particulate phase of cigarette smoke is the likely leading cause (Dalrymple et al., 2018; Haiduc et al., 2020; Zanetti et al., 2019). Because the mechanisms of aerosol generation differ between HTPs and conventional cigarettes, the compositions of HTP aerosols should differ from those of cigarette smoke (Mallock et al., 2019). Therefore, the effect of HTP aerosol on tooth discoloration may differ from that of cigarette smoke.

Particular studies in recent years have assessed the effects of HTP aerosol on tooth discoloration with in vitro techniques. Aerosol from tobacco heating systems 2.2 (THS 2.2) was generated by a smoking machine, and composite resins, artificial denture teeth, bovine teeth, and human premolars were exposed to this aerosol. The effect of THS 2.2 aerosol on the discoloration of these samples was reported to be lower when compared with conventional cigarette smoke (Wang et al., 2022; Zanetti et al., 2019; Zhao et al., 2017). Aerosol from tobacco heating products 1.0 (THP 1.0) was also generated by a smoking machine, and bovine teeth were exposed to this aerosol. Discoloration of bovine teeth by THP 1.0 aerosol was lower when compared with discoloration by conventional cigarette smoke (Dalrymple et al., 2018). These data showed that HTP use as an alternative to conventional cigarettes has the potential to reduce tooth discoloration associated with smoking. However, even though there are some HTPs with various specifications in the market, only a few HTPs, such as THS 2.2 and THP 1.0, have been evaluated. Moreover, previous studies did not investigate the effect of flavor types of the products on tooth discoloration.

**Figure 1:**
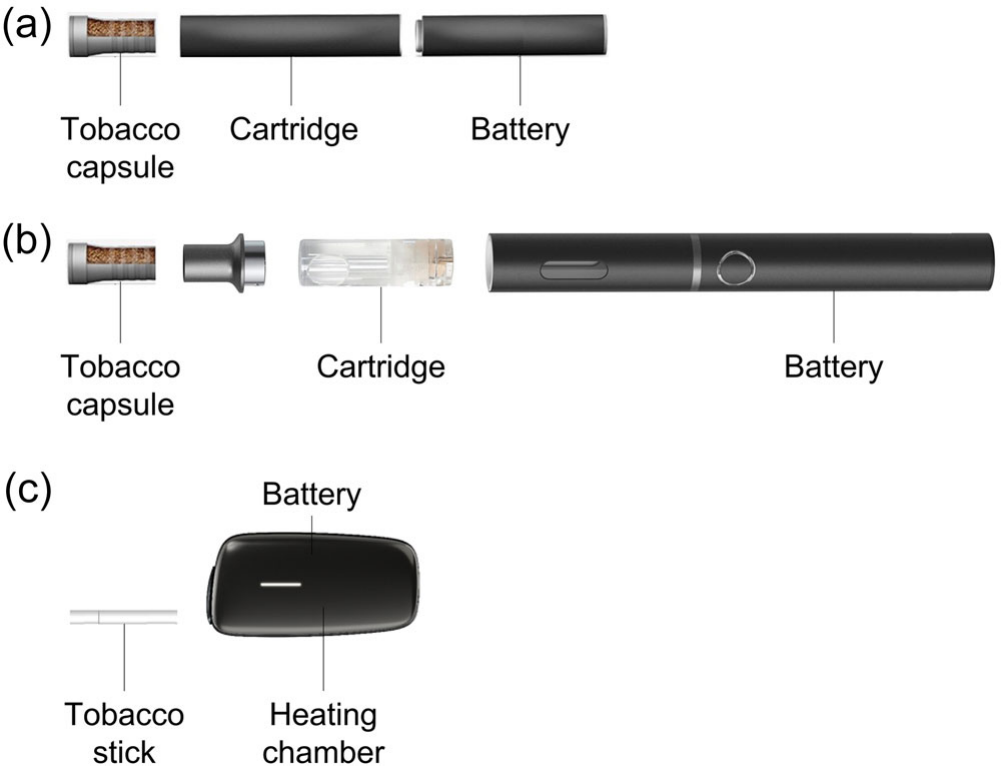
Components of three heated tobacco products used in this study. In‐direct heating tobacco system platform 1.0a (a) and in‐direct heating tobacco system platform 2.0a (b) are composed of a tobacco capsule, cartridge, and battery. Direct heating tobacco system platform 3.0a (c) is composed of a tobacco stick and holder with a battery and heating chamber.

The aim of this study was to assess the tooth discoloration potential of HTPs with different product specifications. We employed three HTPs; in‐direct heating tobacco system platform 1.0a (IT1.0a), in‐direct heating tobacco system platform 2.0a (IT2.0a), and direct heating tobacco system platform 3.0a (DT3.0a). IT1.0a and IT2.0a consist of a battery, cartridge, and tobacco capsule (Figure 1). The cartridge contains liquid composed mainly of propylene glycol and glycerol without nicotine. The aerosol is generated by electrical heating of the liquid, which is passed through the capsule containing granulated tobacco. The user inhales aerosols with flavors and nicotine from the granulated tobacco through thermal distillation at approximately 30–40°C. DT3.0a comprises a tobacco stick and holder with a battery and heating chamber (Figure 1). The user inhales aerosol with flavors and nicotine from the tobacco stick heated directly in the chamber up to a maximum of approximately 295°C. Bovine teeth were exposed to aerosols generated from the three HTPs with different specifications, and enamel discoloration was analyzed and compared with the discoloration of bovine teeth exposed to cigarette smoke. In addition, each HTP was tested with three different flavor types (i.e., regular, menthol, and berry menthol flavors) to investigate the effect of flavor types on the tooth discoloration level.

## 2 MATERIALS AND METHODS

### 2.1 Tobacco products

Kentucky reference 1R6F cigarettes (University of Kentucky) was used as a representative conventional cigarettes. IT1.0a and IT2.0a were obtained from Japan Tobacco Inc. (JT), and DT3.0a was obtained from JT International (Figure 1). IT1.0a is designed to heat granulated tobacco through thermal distillation at approximately 30°C. IT2.0a is similar to IT1.0a, but the temperature of thermal distillation is approximately 40°C. DT3.0a generates aerosol by heating a tobacco stick in a heating chamber up to a maximum of approximately 295°C. Three flavor types (regular, menthol, and berry menthol flavors) were tested for each HTP. Tobacco capsules, cartridges, and tobacco sticks were conditioned at 22 ± 1°C and 60 ± 3% relative humidity for at least 48 h before use, according to the International Organization for Standardization (ISO) 3402:1999 (ISO, 1999).

### 2.2 Preparation of bovine tooth samples

**Figure 2:**
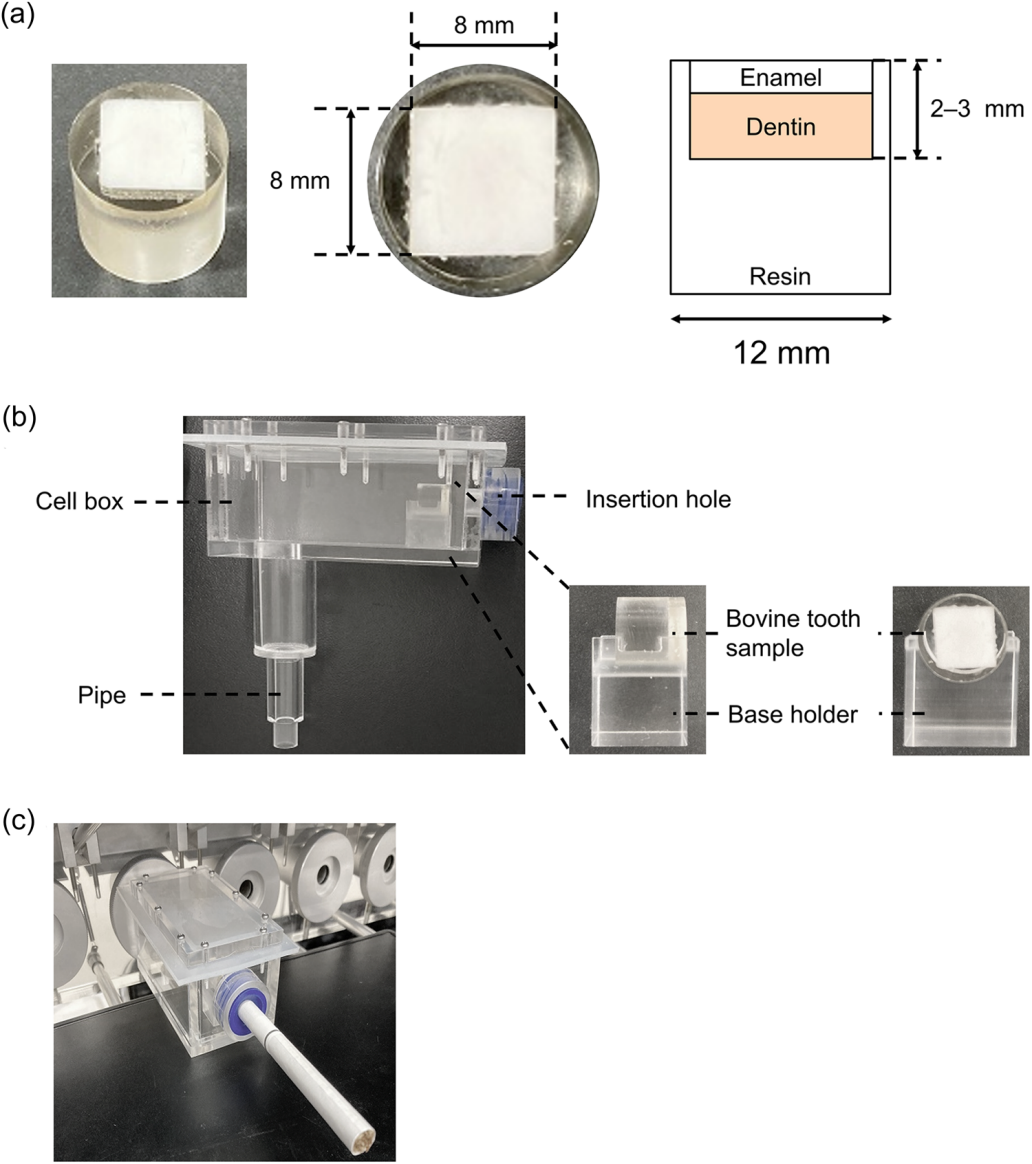
Apparatus for direct exposure of tooth samples to aerosols. (a) Photo and schematic diagram of bovine tooth sample. The tooth block (approximately 8 × 8 mm and 2–3 mm thick) from the central region of the bovine incisor was embedded in a transparent resin substrate (12 mm in diameter). (b) Photo of original exposure module. The module comprises an insertion hall, a small cell box, and a pipe. A bovine tooth sample was placed on the base holder in the box. (c) The module is connected to the SM450RH smoking machine.

The bovine tooth samples were prepared by JFE Techno‐Research Corporation. The bovine incisors were purchased from Tokyo Shibaura Zoki, and approximately 8.0 × 8.0 mm blocks were obtained from the middle portion of the buccal aspect of the bovine tooth crowns (Figure 2a). Each block was embedded in epoxy resin (EpoFix Kit; Struers) using a mold that was 15 mm high with an internal diameter of 12 mm (Figure 2a). After curing the resin, the enamel surface of bovine tooth blocks was polished in a polishing machine using silicon carbide paper #600 (IMT Co., Ltd.), #800 (Struers), and #1200 (Struers). The surface of the blocks was then buffed with diamond abrasive spray (3.0 and 1.0 µm; Struers) and colloidal silica (0.04 µm; Struers) to give the enamel surface a glossy finish. Tooth samples meeting the following criteria were used for exposure: the enamel surface of the bovine block was flat, the bovine block was completely embedded in resin except for the enamel surface, and the thickness of the bovine block was 2–3 mm.

### 2.3 Exposure of bovine tooth samples to aerosols

A bovine tooth sample was placed in the original module for exposure. The module consisted of three parts: an insertion hole for tobacco products, a small cell box with 80 cm^3^ of volume, and a pipe extending downward (Figure 2b). The insertion hole was attached to four labyrinth seals and a neoprene washer, according to ISO 3308:2012 (ISO, 2012) for smoking 1R6F cigarettes. The washer was not used when testing HTPs to avoid the washer absorbing the HTP aerosol.

Before exposure, each tooth sample was incubated in artificial saliva (Pappas et al., 2008) for at least 1 h at 37°C. Each tooth sample was then placed on the base holder in the module (Figure 2b). The module was connected to an SM450RH smoking machine (Cerulean), and test products (1R6F and HTPs) were inserted into the hole of the module (Figure 2c). The enamel surface of the tooth sample faced the end of the tobacco filter. Smoke from a 1R6F cigarette was generated under the ISO intense smoking regime (a 55 mL puff taken over 2 s and repeated every 30 s with 100% vent‐hole blocking) (ISO20778, 2018). Air and aerosols from IT1.0a, IT2.0a, and DT3.0a were also generated under the ISO intense regime but without blocking the vent hole. Aerosols were generated in a test atmosphere of 22 ± 2°C and 60 ± 5% relative humidity according to ISO 3402:1999 (ISO, 1999). One pack each of 1R6F, IT1.0a, IT2.0a, and DT3.0a was used for aerosol generation. The total number of puffs exposed to each tooth sample is summarized in Table 1.

**Table 1:** The total number of puffs exposed to tooth samples.

Group	Product quantities	Number of puffs	Total number of puffs
1R6F	20 cigarettes/pack	8 puffs/cigarette	160 puffs/pack
IT1.0a	5 capsules/pack	70 puffs/capsule	350 puffs/pack
IT2.0a	5 capsules/pack	65 puffs/capsule	325 puffs/pack
DT3.0a	20 sticks/pack	11 puffs/stick	220 puffs/pack
Air	–	70 puffs	350 puffs

Abbreviations: 1R6F, 1R6F cigarette; DT3.0a, direct heating tobacco system platform 3.0a; IT1.0a, in‐direct heating tobacco system platform 1.0a; IT2.0a, in‐direct heating tobacco system platform 2.0a.

After exposure, each tooth sample was stored in 1 mL of phosphate‐buffered saline (pH 7.4; Thermo Fisher Scientific) for 1 h at 37°C. The enamel surface of each tooth sample was then wiped with a paper towel. Each tooth sample was sealed in a 5 mL tube until color measurements. Tooth samples for the assessment of HTPs were sent to JFE Techno‐Research Corporation for color measurements.

### 2.4 Color measurements

The standard Commission Internationale de I'Eclairage (CIELab) color system was adopted for tooth color measurements. The CIELab system is a three‐dimensional color space where L* indicates the black–white (0–100) lightness coordinate, a* indicates the green–red (−*a* = green; +*a* = red) chromaticity coordinate, and b* indicates the blue–yellow (−*b* = blue; +b = yellow) chromaticity coordinate. A CM‐700d spectrophotometer (Konica Minolta Inc.) used for the color measurement was set up with a 10° viewing angle, D65 illuminant, and 3 mm aperture in the SCI mode. Calibration of the CM‐700d was conducted with a white reference tile before every measurement. Before and 2 days after the exposure, the color of each bovine tooth sample was measured at four different points on the enamel surface. L*, a*, and b* values were calculated as means of four measurements. The differences in lightness and chromaticity between before and after aerosol exposure (ΔL*, Δa*, and Δb*) and overall color differences (Δ*E*) of each tooth sample were calculated by the CM‐700d using the following equation: ΔL* = (L* value after exposure) – (L* value before exposure), Δa* = (a* value after exposure) – (a* value before exposure), Δb* = (b* value after exposure) – (b* value before exposure), and Δ*E* = [(ΔL*)^2^ + (Δa*)^2^ + (Δb*)^2^]^1/2^.

### 2.5 Statistical analysis

Six bovine tooth samples were exposed to each condition and means and standard deviations were calculated. A sample size of six was selected based on ISO 28399:2021 (ISO, 2021). Differences in Δ*E* between the five exposure groups (air, 1R6F cigarette smoke, and aerosols from HTPs with three flavors) were analyzed by one‐way analysis of variance (ANOVA) and Tukey's post hoc multiple comparison test using GraphPad Prism software, Version. 9.3.1. (GraphPad Software, http://www.graphpad.com/, RRID:SCR_002798). The significance level was set at *α* = .05.

## 3 RESULTS

### 3.1 Dose‐dependent increase of tooth discoloration by exposure to cigarette smoke

**Figure 3:**
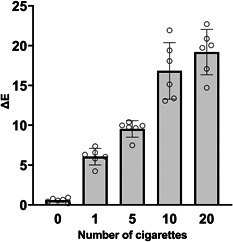
Δ*E* values of bovine tooth samples following exposure to 1R6F cigarette smoke. The bar graphs represent the means and standard deviations of six bovine tooth samples (open circle).

We initially analyzed the tooth discoloration level induced by exposure to 1R6F cigarette smoke to establish conditions for direct aerosol exposure of tooth samples. Exposure was performed with a maximum exposure dose of 20 cigarettes (one pack of 1R6F cigarettes), and discoloration of bovine tooth samples was analyzed by ΔL*, Δa*, and Δb* values. Increasing the number of cigarettes smoked caused a decrease in ΔL* values (Table 2). This result indicates that the lightness of the bovine tooth samples decreased. Δa* and Δb* values increased when the number of cigarettes smoked increased (Table 2). The increase of Δa* indicated that the redness of the bovine tooth samples increased. The increase of the Δb* value means the yellowness of the bovine tooth samples increased. Δ*E* values were calculated from ΔL*, Δa*, and Δb* to yield the overall color change. Δ*E* values increased as the number of cigarettes smoked increased (Figure 3).

**Table 2:** ΔL*, Δa*, and Δb* values (mean ± standard deviation) of bovine tooth samples following exposure to 1R6F cigarette smoke.

	Number of cigarettes smoked				
Parameter	0	1	5	10	20
ΔL*	–0.28 ± 0.28	–2.60 ± 0.65	–4.13 ± 0.72	–7.82 ± 1.83	–10.49 ± 2.19
Δa*	–0.09 ± 0.11	0.14 ± 0.29	0.67 ± 0.32	1.45 ± 0.85	2.83 ± 1.15
Δb*	–0.26 ± 0.43	5.45 ± 0.87	8.55 ± 0.86	14.77 ± 3.22	15.70 ± 2.51

### 3.2 The effect of HTP aerosols on bovine tooth discoloration

The established conditions for direct aerosol exposure were applied to analyze the effect of HTP aerosol on tooth discoloration when compared with tooth discoloration caused by 1R6F cigarette smoke. Each product pack was used to generate aerosols (Table 1). ΔL*, Δa*, and Δb* values of air‐exposed control samples were in the range of ±1.0 (Table 3). Tooth samples exposed to 1R6F cigarette smoke showed a decrease in the ΔL* value and increases in the Δa* and Δb* values compared with the air‐exposed control samples (Table 3). Exposure of bovine teeth samples to aerosols generated from IT1.0a and IT2.0a yielded essentially no change in L*, a*, and b* values regardless of flavor types and their ΔL*, Δa*, and Δb* values were similar to those of air‐exposed control samples (Table 3). After exposure of bovine teeth samples to aerosols from DT3.0a, a decrease in the L* value and an increase in the b* value were observed regardless of flavor type. However, ΔL* and Δb* values of DT3.0a indicated that the changes in L* and b* values were moderate compared with those observed after exposure of samples to 1R6F cigarette smoke (Table 3).

**Table 3:** ΔL*, Δa*, and Δb* values (mean ± standard deviation) of bovine tooth samples following exposure to air, 1R6F cigarette smoke, and each HTP aerosol.

	Air	1R6F	IT1.0a			IT2.0a			DT3.0a		
Parameter	–	–	R	M	BM	R	M	BM	R	M	BM
ΔL*	–0.11 ± 0.34	–8.93 ± 2.14	0.01 ± 0.40	–0.08 ± 0.22	–0.22 ± 0.17	0.23 ± 0.35	–0.36 ± 0.46	–0.29 ± 0.21	–1.58 ± 0.74	–1.55 ± 0.43	–2.39 ± 0.84
Δa*	–0.15 ± 0.07	2.11 ± 0.99	–0.14 ± 0.08	–0.11 ± 0.16	–0.05 ± 0.13	–0.07 ± 0.06	–0.09 ± 0.04	–0.13 ± 0.15	–0.53 ± 0.37	–0.49 ± 0.15	–0.30 ± 0.15
Δb*	0.09 ± 0.25	15.87 ± 1.32	0.12 ± 0.35	0.26 ± 0.27	0.47 ± 0.35	0.19 ± 0.33	0.52 ± 0.81	0.53 ± 0.48	5.14 ± 2.04	5.04 ± 1.18	6.74 ± 1.71

Abbreviations: 1R6F, 1R6F cigarette; BM, berry menthol flavor type; DT3.0a, direct heating tobacco system platform 3.0a; HTP, heated tobacco product; IT1.0a, in‐direct heating tobacco system platform 1.0a; IT2.0a, in‐direct heating tobacco system platform 2.0a; M, menthol flavor type; R, regular flavor type.

**Figure 4:**
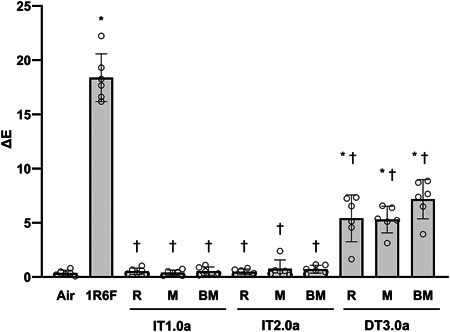
Δ*E* values of bovine tooth samples following exposure to air and aerosols generated from 1R6F cigarettes and three flavors of each HTP. The bar graphs represent the means and standard deviations of six bovine tooth samples (open circle). An asterisk (*) and a dagger (†) indicate a significant difference (*p* < .05) versus the air‐exposed control and 1R6F cigarette smoke‐exposed control, respectively. 1R6F, 1R6F cigarette; BM, berry menthol flavor type; DT3.0a, direct heating tobacco system platform 3.0a; HTP, heated tobacco product; IT1.0a, in‐direct heating tobacco system platform 1.0a; IT2.0a, in‐direct heating tobacco system platform 2.0a; M, menthol flavor type; R, regular flavor type.

Δ*E* values of each product were calculated from ΔL*, Δa*, and Δb* values. Δ*E* values for tooth samples exposed to IT1.0a and IT2.0a were similar to those determined from the air‐exposed control samples and significantly lower than that observed in 1R6F cigarette smoke‐exposed samples regardless of flavor types (Figure 4). Δ*E* values of tooth samples exposed to DT3.0a aerosols were significantly higher than those of air‐exposed control samples but significantly lower than those of 1R6F cigarette smoke‐exposed samples regardless of flavor types (Figure 4). Δ*E* values of each product did not show significant differences among the three flavor types.

## 4 DISCUSSION

We compared the effect of HTP aerosols on tooth discoloration with that of cigarette smoke in this in vitro study. Bovine tooth samples were exposed to aerosols generated from three different HTPs (IT1.0a, IT2.0a, and DT3.0a) and the 1R6F cigarette using a novel direct aerosol exposure system. Three flavor types were tested for each HTP. The results indicated that 1R6F cigarette smoke caused discoloration of bovine tooth samples. For the three tested HTPs, aerosols from IT1.0a and IT2.0a caused minimal bovine tooth sample discoloration, regardless of flavor type. Aerosols from DT3.0a caused slight discoloration of bovine tooth samples, as indicated by the Δ*E* value, but the level was significantly lower than the discoloration caused by 1R6F cigarette smoke regardless of flavor type.

Reproducing the situation in the mouth of a user is desirable when investigating the effect of HTP aerosols on tooth discoloration in vitro. Bovine teeth were used in this study as surrogates of human teeth because they have similar microhardness values and chemical composition to human teeth (Arango‐Santander et al., 2020; Teruel et al., 2015; Yassen et al., 2011). Exposure of bovine tooth samples to aerosols was performed with consideration of the following three points. First, aerosols generated from HTPs should be exposed to tooth samples directly. This point is important in in vitro toxicity testing of HTP aerosols, and several exposure systems have been used for exposing air‐liquid interface cultured cells (Ishikawa et al., 2018; Thorne et al., 2018; Zanetti et al., 2017). Some of these systems have been used in previous studies to expose tooth samples instead of cells. Second, the tooth sample should be placed proximal to the tobacco filter. Aerosols generated from cigarettes and HTPs can adhere to the aerosol flow path. Therefore, the aerosol flow path should be as short as possible to avoid aerosol loss in the exposure system. Placing the tooth sample with the enamel surface facing the end of the tobacco filter is optimal. Lastly, an appropriate smoking regime should be used for aerosol generation. Smoking regimes that control the puff volume and smoke/aerosol stream should be used when generating aerosols by a smoking machine (Zhao et al., 2017). The exposure setup used in this study was designed to meet these three points.

The exposure setup was used to detect discoloration of tooth samples caused by cigarette smoke. Cigarette smoke is a widely known factor that causes tooth discoloration (Joiner et al., 2008; Watts & Addy, 2001), and brown pigments in the tar are the main coloring agents (Haiduc et al., 2020; Miranda et al., 2011). Our results showed a dose‐dependent increase of ΔL*, indicating that bovine tooth samples darkened following exposure to 1R6F cigarette smoke. Dose‐dependent increases of Δa* and Δb* were also observed, indicating that bovine tooth samples became red and yellow, respectively. The results of 1R6F cigarette smoke exposure using our novel system successfully reproduced the staining characteristics derived from the deposition of the pigments in the tar.

For comparing the effect on tooth discoloration of the aerosols generated from HTPs with that from 1R6F cigarette smoke, the exposure dose of each product was set based on the amount consumers use each product in a day. We used smoke generated from one pack of 1R6F cigarettes (20 cigarettes) because the global average daily cigarette consumption is 16.5 (Organisation for Economic Cooperation and Development, 2021). We also used aerosols generated from one pack of the DT3.0a (20 sticks) because the daily consumption of stick‐type HTPs was shown in a recent clinical study to be almost the same as that of cigarettes (Yuki et al., 2022). For IT1.0a and IT2.0a, one pack was used because the number of capsules in one pack (five capsules) is close to the daily consumption of these types of products (Sakaguchi et al., 2021; Yuki et al., 2022). The number of puffs per product was set as the maximum number of puffs based on the product specifications. The number of puffs for air exposure was adjusted to that of IT1.0a, which has the highest number of puffs.

The results examining three HTPs indicated that aerosols from these HTPs showed reduced tooth discoloration potential compared with 1R6F cigarette smoke, whereas the level of reduction differed among the HTPs. Exposure of bovine tooth samples to aerosols from IT1.0a and IT2.0a showed hardly any change in tooth color, and Δ*E* values were similar to the Δ*E* value obtained for the air‐exposed control samples. However, bovine tooth samples exposed to the DT3.0a aerosol yielded a higher Δ*E* value when compared with the air‐exposed control samples. This difference may arise from the different heating temperatures of tobacco used. DT3.0a heats tobacco up to a maximum of approximately 295°C for aerosol generation, and this temperature is higher when compared with IT1.0a and IT2.0a (30–40°C heating by thermal distillation). Several studies have indicated that the heating temperature of tobacco is an important factor for mainstream aerosol chemistry (Forster et al., 2018; Schaller et al., 2016; Takahashi et al., 2018), and it has been reported that higher heating temperatures lead to greater kinds and amounts of components generated (Mallock et al., 2019). The results with DT3.0a support previous studies using THS 2.2, which heats tobacco sticks at 350°C. In those studies, although it is difficult to directly compare the Δ*E* value of THS 2.2 with that of DT3.0a obtained herein because of different experimental conditions (e.g., exposure dose, exposure duration, and with/without brushing), THS 2.2 was reported to cause a slight change in the Δ*E* value of composite resin and bovine and human tooth samples after exposure to the aerosol (Haiduc et al., 2020; Zanetti et al., 2019).

In addition to differences in product specifications, we also investigated the effect of flavor types on tooth discoloration. Several studies have investigated the effect of HTP aerosol on tooth discoloration (Dalrymple et al., 2018; Wang et al., 2022; Zanetti et al., 2019; Zhao et al., 2017). However, a single representative flavor was tested in these studies. We selected regular, menthol and berry menthol flavors for each HTP product. The results indicated that the discoloration level was similar regardless of flavor types in the same product. A previous study of e‐cigarettes showed that e‐liquids with flavor (tobacco or menthol flavor) decreased the yellowness of the enamel in comparison with a flavor‐neutral e‐liquid (Pintado‐Palomino et al., 2019). However, this study used e‐liquids with color and suggested that chromas in the e‐liquid affect the whiteness of enamel by decreasing yellowness. IT1.0a and IT2.0a have cartridges containing liquid, and this liquid is vaporized and used for heating granulated tobacco. This liquid is mainly composed of propylene glycol and glycerol and is colorless. Tooth discoloration is caused by the deposition of chemicals that have color. Thus, using liquid without color may represent an approach to reduce changes in tooth color.

The in vitro system used in this study was designed without brushing the surface of the tooth samples with toothpaste. It is reported that oral hygiene is a crucial factor that affects tooth discoloration caused by staining on the salivary pellicle by smoking (Franzò et al., 2010; de Geus et al., 2018; Terézhalmy et al., 2004). In addition, tooth discoloration is expected to develop as exposure to cigarette smoke or HTP aerosols increases. Therefore, a study introducing long‐term repeated exposure and tooth brushing is the next step toward providing data on tooth discoloration of aerosols generated from HTPs under conditions that are close to real‐life conditions.

## 5 CONCLUSION

We have developed a direct aerosol exposure system for examining bovine tooth samples. The system successfully reproduced tooth discoloration following exposure to 1R6F cigarette smoke. The aerosols generated from HTPs (IT1.0a, IT2.0a, and DT3.0a) have a reduced effect on tooth discoloration when compared with tooth discoloration caused by 1R6F cigarette smoke, regardless of the tested flavor types. This reduction may be attributable to the decrease in chemical constituents derived from the combustion of tobacco leaves that cause discoloration of tooth samples. Further studies are required to clarify the significance of our in vitro testing data.

## AUTHOR CONTRIBUTIONS

*Supervised the research, designed the research, carried out the experiments, analyzed and interpreted data, and wrote the article*:Takeshi Kurachi. *Designed the research, conducted the experiments, inspected and interpreted data, and reviewed the article*: Shinnosuke Chuman. *Designed the research, examined and interpreted data, and reviewed the article*: Takuya Suzuki. *Designed the research, examined and interpreted data, and reviewed the article*: Terushige Kubota. *Helped supervise the research, supervised the writing, and critically revised the article*: Shinkichi Ishikawa. All authors approved the final version of the article.

## CONFLICT OF INTEREST STATEMENT

The authors declare no conflict of interest.

## Data Availability

The data sets used and/or analyzed during the current study are available from the corresponding author upon reasonable request.
